# Complete Genome Sequence of a Pathogenic Genotype 1 Subtype 3 Porcine Reproductive and Respiratory Syndrome Virus (Strain SU1-Bel) from Pig Primary Tissue

**DOI:** 10.1128/genomeA.00340-15

**Published:** 2015-05-21

**Authors:** Zen H. Lu, Alison D. Wilson, Xinglong Wang, Jean-Pierre Frossard, Tomasz Stadejek, Alan L. Archibald, Tahar Ait-Ali

**Affiliations:** a The Roslin Institute and Royal (Dick) School of Veterinary Studies, University of Edinburgh, Easter Bush, United Kingdom; b Virology Department, Animal and Plant Health Agency (APHA), Weybridge, Addlestone, United Kingdom; c Department of Pathology and Veterinary Diagnostics, Faculty of Veterinary Medicine, Warsaw University of Life Sciences, Warsaw, Poland

## Abstract

We report here the complete genome of the pathogenic eastern European subtype 3 porcine reproductive and respiratory syndrome virus (PRRSV) strain SU1-Bel, sequenced directly from a pig lymph node. While sharing substantial sequence similarity with other subtype 3 strains, SU1-Bel is found to harbor unique indels and contain putative novel subgenomic RNAs.

## GENOME ANNOUNCEMENT

Porcine reproductive and respiratory syndrome virus (PRRSV) is the etiologic agent of a swine disease causing immense losses to the pig industry since the late 1980s. This positive-stranded RNA arterivirus exists in two distinct genotypes, European type 1 (PRRSV-1) and North American type 2 (PRRSV-2). The emergence of highly pathogenic PRRSV-2 strains in China has resulted in the death of millions of animals and, more recently, pathogenic PRRSV-1 subtype 3 strains capable of inducing fatal infections in pigs have also been isolated in Belarus (1–4). However, increasing observations suggest that different pathogenic PRRSV-1 strains may elicit different host responses via dissimilar routes. For example, PRRSV-1 subtype 3 strain Lena showed enhanced replication *in vivo* (1–3, 5, 6), whereas SU1-Bel, another subtype 3 strain, caused inflammatory response without a marked increase in replication (1). A whole-genome comparison of these strains is therefore warranted.

We were able to assemble the complete genome of SU1-Bel without any prior knowledge of the sequence from the primary tissue of a pig experimentally infected with the virus (1). Total RNA was isolated from tracheobronchial lymph nodes 3 days postinfection (p.i.), reversed transcribed, and a 101-bp paired-end RNA-sequencing (RNA-seq) library was constructed, as described previously (7). Sequencing was performed on the Illumina HiSeq 2500 platform at Edinburgh Genomics (United Kingdom) (http://genomics.ed.ac.uk). Contaminating pig and adapter-containing sequencing reads were discarded, and low-quality (Phred score, <20) reads were trimmed before the remaining reads were assembled *de novo* using Velvet (version 1.2.09) (8) at multiple *k*-mers. The full-length genome sequence was then obtained by merging the Velvet contigs using the Lasergene SeqMan Pro (version 10.1). This genomic sequence was confirmed by remapping the reads back to the consensus sequence using BWA-MEM (version 0.7.10) (9).

The 14,958-nucleotide (nt) SU1-Bel genome shares a substantial sequence similarity with other PRRSV-1 strains, with 79% and 88% similarity with the prototypical strain Lelystad (LV) and strain Lena, respectively. However, not only is the SU1-Bel genome the shortest among the three, it also harbors unique indels. While the highest variability clusters on the *Nsp2* gene, small insertions, compared with that of strain Lena, are present in both *Nsp1* and the 3′ untranslated region (UTR). The significance of these variations remains to be investigated.

Consistent with other reported PRRSV strains, an analysis of the genome shows that SU1-Bel contains 10 open reading frames (ORFs), which can be translated into all the known PRRSV (poly)proteins. Interestingly, in addition to the full-length genomic RNA transcripts, contigs from the assembled reads strongly suggest the transcription of multiple novel subgenomic RNAs by SU1-Bel infecting the lymph nodes. Some of them are predicted to encode novel proteins not previously described in other PRRSV-1 strains.

To our knowledge, this is the first complete genome sequence of a subtype 3 PRRSV-1 determined directly from primary tissue. Together with that of strain Lena, the SU1-Bel sequence will enable us to gain further understanding into the genetic diversity, epidemiology, and pathogenesis of PRRSV-1 subtype 3 strains.

### Nucleotide sequence accession number. 

The complete genome sequence of SU1-Bel was deposited in GenBank with the accession no. KP889243.
